# Comparative Assessment of Color Stability in Four Commercial Clear Aligner Materials Exposed to Common Beverages: An In Vitro Study

**DOI:** 10.7759/cureus.98482

**Published:** 2025-12-04

**Authors:** Sameer Narkhede, Paritosh Rao, Veera Sawant, Shreedaay Kambli, Nikita Garg, Shreyas P Shah, Sanpreet S Sachdev

**Affiliations:** 1 Orthodontics and Dentofacial Orthopedics, DY Patil University School of Dentistry, Navi Mumbai, IND; 2 Orthodontics, Arte Aligners, Mumbai, IND; 3 Orthodontics, Dr. S.S. Tantia Dental College, Hospital and Research Center, Sri Ganganagar, IND; 4 Oral Medicine and Radiology, Bharati Vidyapeeth (Deemed to be University) Dental College and Hospital, Navi Mumbai, IND; 5 Oral Pathology and Microbiology, Bharati Vidyapeeth (Deemed to be University) Dental College and Hospital, Navi Mumbai, IND

**Keywords:** clear aligner, colour stability, petg, polyurethane, spectrophotometry

## Abstract

Background

Clear aligner therapy (CAT) has become a popular esthetic alternative to fixed orthodontic appliances, driven by advances in polymer science and digital manufacturing. However, staining and color instability remain significant clinical concerns, especially given patient non-compliance with recommended beverage restrictions. Limited comparative evidence exists regarding how different aligner polymers respond to common chromogenic beverages.

Aim

To evaluate and compare the color stability of four commercially available clear aligner materials, Invisalign® (polyurethane; Align Technology Inc., Tempe, Arizona, United States), ClearCorrect™ (polyethylene terephthalate glycol (PETG); Round Rock, Texas, United States), Illusion® (multilayer polyester blend; Laxmi Dental Ltd., Mumbai, India), and Graphy TC-85® (photocurable oligomer; Graphy Inc., Geumcheon-gu, Seoul, South Korea), following immersion in tea, coffee, cola, and red wine over a two-week period.

Materials and methods

This laboratory-based in vitro study used 40 aligner specimens (10 per brand), sectioned into standardized 10×10 mm samples. Specimens were randomly assigned to five immersion media (tea, coffee, cola, red wine, and distilled water). Color measurements (L*, a*, b*) were recorded at baseline, 24 hours, seven days, and 14 days using a VITA Easyshade® V (VITA Zahnfabrik H. Rauter GmbH & Co. KG, Germany) spectrophotometer. Total color change (ΔE) was calculated using the CIELAB formula. Data were analyzed using repeated-measures ANOVA with Tukey’s post hoc tests (α=0.05).

Results

All aligners exhibited clinically acceptable baseline ΔE values (<3.3). Coffee and cola produced the highest discoloration across most materials. ClearCorrect™ and Illusion® demonstrated the greatest susceptibility, exceeding perceptible thresholds within 24 hours. Invisalign® showed moderate, beverage-independent staining, while Graphy TC-85® remained stable against acidic beverages but showed delayed tea-related discoloration. Time and beverage type significantly influenced ΔE values (p<0.001).

Conclusion

Color stability of clear aligners is strongly governed by polymer chemistry and beverage characteristics. PETG and multilayer polyester blends stain rapidly, polyurethane discolors moderately, and photocurable oligomers display beverage-specific behavior. Material-specific guidance and reinforced patient instructions are essential to maintain esthetic longevity of aligners.

## Introduction

Clear aligner therapy (CAT) has evolved from a niche orthodontic adjunct into a mainstream alternative to conventional fixed appliances (FAs), propelled by significant advancements in biomaterials, biomechanics, and digital manufacturing technologies such as computer-aided design (CAD) and computer-aided manufacturing (CAM). The nearly invisible appearance of aligners, coupled with increased patient awareness through direct-to-consumer marketing and social media engagement, has particularly attracted adult and young adult populations seeking discreet and comfortable orthodontic treatment options [1]. This growing preference has translated into a remarkable global market expansion, estimated at USD 3.1 billion in 2021 and projected to surpass USD 11 billion by 2027, representing a compound annual growth rate of nearly 13% [2]. Such figures highlight not only the commercial success of aligner systems but also their widespread clinical acceptance as an esthetic and lifestyle-compatible orthodontic modality.

In principle, CAT employs a series of transparent trays, each designed to induce controlled, incremental tooth movement. These trays are either thermoformed from prefabricated amorphous thermoplastic sheets or additively manufactured using 3D printing technologies with photopolymer resins [3]. While the clinical effectiveness of aligners depends on mechanical parameters such as force delivery, attachment configuration, and patient compliance, the chemical composition and physical behavior of the base polymer are equally crucial in determining their optical stability, flexibility, and wear resistance [4-7]. The concept of sequential removable appliances dates back to Kesling’s 1945 innovation of the rubber tooth positioner, which demonstrated the feasibility of progressive alignment [8]. This concept evolved further with Nahoum’s introduction of clear thermoplastic appliances in 1964 and Ponitz’s development of vacuum-formed retainers in 1971, laying the foundation for the modern clear aligner era [9,10]. The digital revolution arrived with the launch of Invisalign® (Align Technology Inc., Tempe, Arizona, United States) in 1998, which integrated 3D scanning, CAD-based treatment planning, and stereolithographic modeling to produce patient-specific polyurethane aligners, marking a transformative shift in orthodontic practice [11,12].

Modern aligner systems vary substantially in terms of their polymer composition and manufacturing processes. Most thermoformed systems, such as Invisalign® and ClearCorrect™ (Round Rock, Texas, United States), utilize amorphous thermoplastic polymers like polyurethane and polyethylene terephthalate glycol (PETG) for their high transparency, elasticity, and ease of thermoforming [13,14]. Differences in thermoforming versus direct 3D printing workflows, including the accuracy of printed models and device fabrication, may further influence the fit and surface behavior of aligners, as highlighted by recent investigations on printed models and clear aligner manufacturing [15,16]. Others employ co-polyester blends or multilayer laminates to enhance mechanical strength and optical clarity, while novel directly 3D-printed aligners such as Graphy TC-85® (Graphy Inc., Geumcheon-gu, Seoul, South Korea) have emerged as next-generation materials, offering greater geometric precision, uniform thickness, and reduced production steps [17,18]. Despite these advancements, maintaining optical transparency remains a significant challenge. The amorphous nature of these polymers, essential for translucency, also predisposes them to chromogenic sorption and absorption, making them susceptible to staining from dietary pigments, acids, and tannins.

Although clinicians instruct patients to remove aligners before consuming any beverage other than water, clinical surveys have reported widespread non-compliance, with many patients consuming tea, coffee, cola, or wine while wearing their trays [19]. This habitual exposure to chromogenic beverages leads to the accumulation of surface pigments and potential internal discoloration, compromising the esthetic advantage that defines aligner therapy. Several in vitro studies have demonstrated that pigmented beverages such as coffee, tea, cola, and red wine can induce perceptible color changes within just a few days of exposure, particularly in polyurethane- and PETG-based aligners [19,20]. These color shifts are not merely superficial but can result from the diffusion of pigment molecules into the polymer matrix or from surface degradation that enhances light scattering.

The molecular structure of the polymer strongly governs its staining susceptibility. Polyurethane, composed of alternating soft and hard segments, contains polar urethane groups that form hydrogen bonds with hydrophilic stains such as tannins, leading to gradual, cumulative discoloration [21]. PETG, on the other hand, exhibits a less ordered molecular structure and possesses ester linkages that are vulnerable to hydrolysis under acidic conditions. This allows deeper penetration of colored and acidic compounds, producing more rapid and pronounced discoloration [22]. Furthermore, factors such as beverage pH, temperature, and exposure duration contribute to variations in color stability among aligner materials. Environmental factors, like brushing habits, salivary flow, and biofilm accumulation, further accentuate staining under clinical conditions, although these effects are difficult to replicate in laboratory studies. Despite the growing body of evidence, most previous investigations have focused on individual aligner brands or single-material comparisons, limiting the ability to draw clinically relevant conclusions across the diverse range of currently available systems. There is, therefore, a pressing need for direct comparative analyses evaluating multiple materials under standardized conditions to determine which combinations of polymer type and beverage exposure are most detrimental to long-term esthetics.

The present study aims to address this gap by systematically evaluating and comparing the color stability of four widely used clear aligner materials, including Invisalign® (polyurethane), ClearCorrect™ (PETG), Illusion® (multilayer polyester blend; Laxmi Dental Ltd., Mumbai, India), and Graphy TC-85® (photocurable oligomer), following immersion in commonly consumed staining beverages including tea, coffee, cola, and red wine. By employing objective spectrophotometric analysis over defined exposure intervals, this investigation seeks to elucidate the interaction between aligner polymer chemistry and beverage-induced discoloration. We hypothesized that PETG and multilayer thermoformed aligners would exhibit greater beverage-induced discoloration than polyurethane and the directly 3D-printed TC-85 resin over 14 days of immersion. The findings are expected to provide clinically relevant insights to guide material selection, improve patient dietary counseling, and help preserve the esthetic longevity that defines successful clear aligner therapy.

## Materials and methods

The present in vitro laboratory study was conducted jointly in the Department of Orthodontics and the institutional biomaterials laboratory. All procedures adhered to the ethical standards of the Declaration of Helsinki and were approved as exempt research by the Institutional Ethics Committee (IEC/ORTHO/2025/004).

Sample size determination

Sample size estimation was performed using OpenEpi (version 3.01) using the formula for comparing two independent means based on the reference values reported by Olteanu et al. [23]. The calculation used mean ΔE values of 1.28 and 2.71, standard deviations of 0.97 and 1.26, and an α error of 0.05 (two-tailed), β=0.20 (80% power), and an equal allocation ratio (κ=1). Using these parameters, the required number of specimens per group was determined to be 10. The formula incorporated the standard deviations of the two groups, the expected mean difference (Δ=−1.43), the allocation ratio κ, and Z-scores for 95% confidence (Z₁-α/2=1.96) and 80% power (Z₁-β=0.84). A total sample size of 40 specimens, comprising 10 per aligner brand, met the calculated requirement while providing balanced representation across staining media.

Aligner materials

Four commercially available clear aligner systems were selected to represent different polymer categories: Invisalign® (polyurethane), ClearCorrect™ (PETG), Illusion® (multilayer polyester blend), and Graphy TC-85® (a photocurable oligomer approved for direct 3D-printed aligners). Unused maxillary trays from the initial treatment series were obtained directly from their respective manufacturers. To minimize variability arising from in-house thermoforming or printing, only manufacturer-fabricated, unused clinical trays from the initial treatment series were used, a strategy consistent with recent work highlighting the impact of model fabrication and printing workflows on aligner-related accuracy [15]. All aligners were stored in double-sealed packaging at 22°C in the dark to prevent premature thermal or photochemical alteration.

Specimen preparation

Each aligner tray was sectioned into standardized 10×10 mm squares using a water-cooled diamond saw to ensure uniform and reproducible flat surfaces. Sections were harvested from the buccal region of the tray, and samples exhibiting defects, such as air bubbles, cracks, or distortion, were excluded under ×2.5 magnification. The thickness of each prepared segment was measured with a calibrated digital caliper (Mitutoyo, Japan) to the nearest 0.01 mm to document material consistency. The thickness of each segment was measured at three points with a calibrated digital caliper and remained within the manufacturer-specified range of 0.75 to 0.8 mm for each system.

Grouping and randomization

From each aligner brand, specimens were randomly assigned to one of five immersion media, including tea, coffee, red wine, cola, or distilled water, resulting in two samples per brand per solution, for a total of 40 specimens. Randomization was performed using computer-generated random numbers in Microsoft Excel (v2019, Microsoft Corporation, Redmond, Washington, USA), with allocation codes concealed in opaque envelopes. The examiner performing spectrophotometric measurements remained blinded to both aligner type and immersion medium to minimize assessment bias.

Preparation of staining media

Standardized staining solutions were freshly prepared each day. Black tea was prepared by simmering 4 g of loose-leaf tea in 100 mL distilled water for five minutes and filtering through a 0.45-µm membrane. Coffee was prepared by dissolving 3 g of spray-dried coffee in 100 mL of distilled water heated to 90°C, followed by filtration. Commercial red wine (13% v/v ethanol) was used without dilution, and cola was degassed ultrasonically for 30 minutes to eliminate carbonation. All media were transferred to amber borosilicate containers and maintained at 37±1°C to simulate intraoral temperature.

Immersion protocol

**Figure 1:**
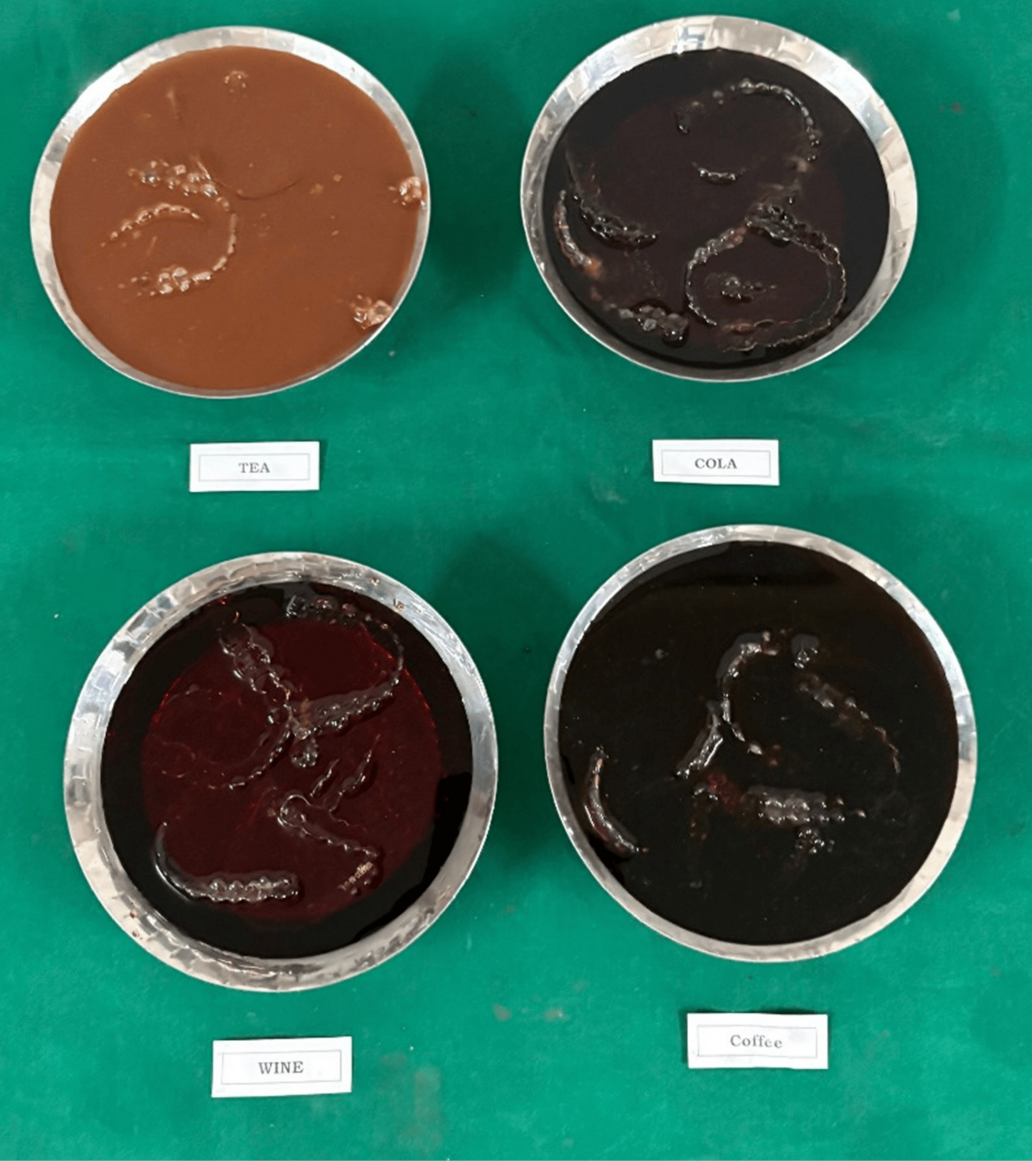
Aligner samples immersed in different solutions

Specimens were fully immersed in their assigned media (Figure 1) and placed in a shaking incubator operating at 70 rpm and 37°C. Baseline color values (T₀) were recorded after one minute of immersion using a calibrated spectrophotometer. The staining solutions were replaced every 24 hours to maintain chromogenic stability. All samples were stored individually in containers to avoid cross-staining. Solutions were checked daily to prevent bacterial overgrowth. Containers in which the aligners were stored were covered to prevent evaporation or moisture contamination. pH was monitored because acidic solutions can alter material properties.

Color measurement

**Figure 2:**
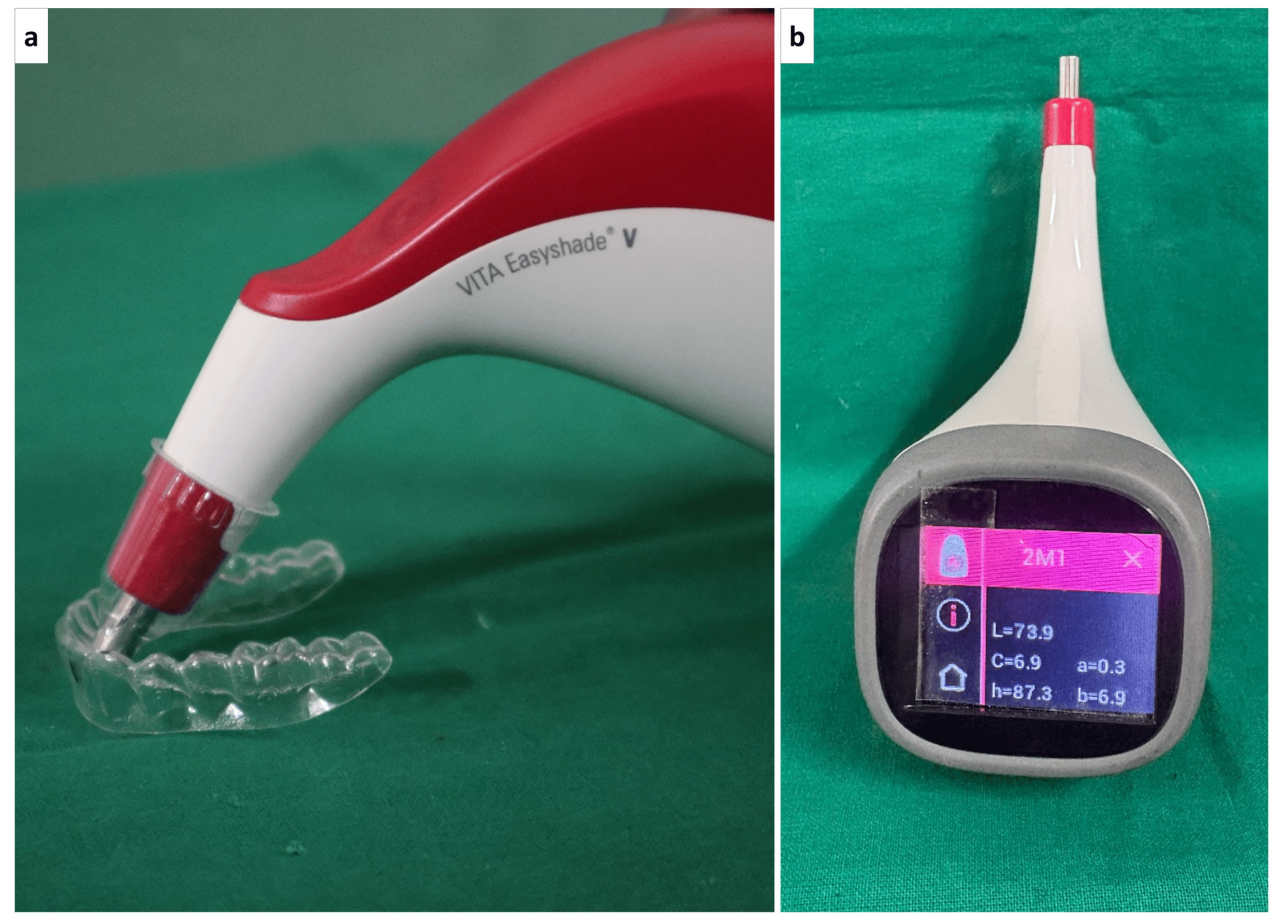
(a) Digital spectrophotometer placed at the center of the aligner; (b) colorimetric readings obtained in the device

Color measurements were repeated at 24 hours (T₁), seven days (T₂), and 14 days (T₃). Before each reading, specimens were rinsed with distilled water for 30 seconds, blotted gently, and air-dried for 60 seconds. Color evaluation was performed using a VITA Easyshade® V (VITA Zahnfabrik H. Rauter GmbH & Co. KG, Germany) spectrophotometer positioned perpendicular to the specimen’s central region (Figure 2). For each time point, three measurements were taken with the specimen rotated 120° between readings, and the mean value was used for analysis. Before each measurement session, the VITA Easyshade® V device was calibrated according to the manufacturer’s instructions using the supplied white reference standard.

Commission Internationale de l'Éclairage Lab (CIELAB) parameters (L*, a*, b*) were recorded, and total color change (ΔE) was calculated using the formula

$∆E=\sqrt{(∆L*)^{2}+ (∆a*)^{2}+(∆b*)^{2}}$

Wherein the L* axis comprised a gray scale with values from 0 (black) to 100 (white). The L* value correlates with the level of pigmentation of an individual. The a* axis represents the red-green axis; positive and negative a* values correspond to red and green, respectively, which correlate with erythema. The b* represents the yellow/blue axis; positive and negative b* values correspond to yellow and blue, respectively, and correlate with pigmentation and tanning. The outcome ∆E is a single number representing the "distance" between two colors. A value of ΔE* ≥ 3.3 was considered clinically perceptible.

Reliability assessment

To verify measurement consistency, five randomly selected specimens were re-measured after one hour by two independent examiners. Intraclass correlation coefficients (ICCs) were calculated, and values exceeding 0.90 were interpreted as indicating excellent intra- and interobserver reliability.

Outcome variables

The primary outcome was the mean total color difference (ΔEab) for each aligner material in each staining medium at T₁, T₂, and T₃. Secondary outcomes included changes in individual CIELAB axes (ΔL*, Δa*, Δb*), which provided additional information on the nature and direction of discoloration.

Statistical analysis

Data distribution was evaluated using the Shapiro-Wilk test. A three-way repeated-measures ANOVA (factors: aligner brand, staining medium, and time) was applied to determine main effects and interactions, with Greenhouse-Geisser correction used when sphericity assumptions were violated. Post hoc pairwise comparisons were conducted using Tukey’s honestly significant difference (HSD). When parametric assumptions were not met, non-parametric tests (Kruskal-Wallis with Dunn-Bonferroni correction) were performed. Statistical significance was set at α=0.05. All analyses were carried out using SPSS Statistics version 28.0 (IBM Corp., Armonk, NY).

## Results

A total of 40 specimens (10 per aligner brand) completed the staining protocol without loss or fracture. Shapiro-Wilk testing confirmed normal distribution for all ΔE data, permitting parametric analyses. Repeated-measures ANOVA showed significant effects of beverage (p<0.001), time (p<0.001), and their interaction (p<0.001) for every aligner, so results are reported by time point.

Baseline (before immersion)

All brands exhibited ΔE values below the clinical threshold of 3.3, indicating essentially imperceptible discoloration at the outset (Table 1). Beverage had no effect on Illusion or Graphy (p>0.05). By contrast, Invisalign and ClearCorrect already showed material-specific susceptibility: coffee and cola produced higher baseline ΔE than wine for Invisalign (p<0.001), and both beverages out-performed tea, coffee, and wine for ClearCorrect (p<0.001).

**Table 1:** Comparison of baseline ∆E values of the aligners

Baseline ∆E	Beverage	N	Minimum	Maximum	Mean	Std. Deviation	F	p Value
Illusion	Tea	10	1.07	3.02	1.674	0.631	2.518	0.073
	Coffee	10	0.97	1.38	1.149	0.146		
	Cola	10	1.09	1.79	1.456	0.252		
	Wine	10	0.00	2.23	1.233	0.635		
Graphy	Tea	10	1.45	2.89	2.074	0.411	2.518	0.073
	Coffee	10	1.26	3.00	2.116	0.640		
	Cola	10	1.56	2.17	1.834	0.198		
	Wine	10	0.00	0.92	0.604	0.305		
Invisalign	Tea	10	1.53	2.23	2.062	0.225	15.078	0.000
	Coffee	10	2.22	3.12	2.800	0.280		
	Cola	10	2.11	2.72	2.480	0.235		
	Wine	10	0.00	3.04	1.150	1.084		
ClearCorrect	Tea	10	4.12	11.31	7.970	2.678	7.997	0.000
	Coffee	10	7.91	11.21	9.300	1.109		
	Cola	10	9.21	10.51	9.784	0.439		
	Wine	10	0.00	10.51	5.106	3.672		

After 24 hours of immersion

Early staining intensified but followed distinct brand-specific patterns (Table 2). Coffee produced the greatest shift in Illusion (mean ΔE=13), whereas cola dominated in ClearCorrect (mean ΔE=14). Graphy remained comparatively color-stable, recording its highest ΔE (<8) with coffee. Invisalign showed no beverage-related differences at this time point (p>0.05). Across all brands, wine caused the least perceptible change.

**Table 2:** Comparison of discoloration of the aligners stained after 24 hours of immersion

∆E After 24 Hours of Immersion	Beverage	N	Minimum	Maximum	Mean	Std. Deviation	F	p Value
Illusion	Tea	10	0.00	10.09	8.0040	2.95015	6.921	0.001
	Coffee	10	13.00	14.24	13.3440	0.41738		
	Cola	10	3.74	21.18	10.7820	6.31892		
	Wine	10	6.40	7.57	6.8570	0.35755		
Graphy	Tea	10	4.03	6.34	5.1250	0.88701	180.458	0.000
	Coffee	10	7.14	7.69	7.3960	0.16352		
	Cola	10	0.75	2.57	1.9590	0.53303		
	Wine	10	3.83	4.33	4.1500	0.17224		
Invisalign	Tea	10	3.28	19.41	10.4790	6.05274	2.119	0.115
	Coffee	10	7.10	8.47	7.8750	0.45809		
	Cola	10	8.55	9.99	9.3940	0.45853		
	Wine	10	5.49	8.89	7.3560	1.17958		
ClearCorrect	Tea	10	3.26	7.27	5.0580	1.40460	102.686	0.000
	Coffee	10	4.16	6.23	4.9860	0.56135		
	Cola	10	11.28	16.31	13.6540	1.86692		
	Wine	10	3.08	6.22	4.6910	1.29589		

After seven days of immersion

Time magnified the beverage effect (Table 3). Cola displaced coffee as the strongest chromogen for Illusion and ClearCorrect, pushing mean ΔE beyond 29 and 20, respectively. Graphy was most vulnerable to coffee (mean ΔE=15) but remained resistant to cola (mean ΔE=5). Invisalign again demonstrated a moderate, evenly distributed response (ΔE=13-18), although cola was now significantly higher than wine (p<0.01).

**Table 3:** Comparison of discoloration of the aligners stained after 7 days of immersion

∆E After 7 Days of Immersion	Beverage	N	Minimum	Maximum	Mean	Std. Deviation	F	p Value
Illusion	Tea	10	10.43	15.84	13.5190	2.02986	584.190	0.000
	Coffee	10	16.65	18.77	17.8450	0.77034		
	Cola	10	28.89	29.34	29.0850	0.17024		
	Wine	10	9.64	10.00	9.8750	0.10617		
Graphy	Tea	10	5.86	8.04	7.0930	0.84782	753.785	0.000
	Coffee	10	14.66	16.31	15.3350	0.57400		
	Cola	10	4.94	5.33	5.1280	0.13147		
	Wine	10	6.70	7.24	7.0490	0.18046		
Invisalign	Tea	10	5.59	28.10	13.3980	6.53538	6.895	0.001
	Coffee	10	12.04	15.17	13.1710	1.13595		
	Cola	10	18.12	18.87	18.5070	0.24432		
	Wine	10	12.33	12.79	12.5510	0.16299		
ClearCorrect	Tea	10	4.23	5.82	4.6240	0.45834	7574.771	0.000
	Coffee	10	4.65	5.35	4.9900	0.21924		
	Cola	10	20.14	20.56	20.3560	0.15472		
	Wine	10	4.36	4.96	4.6470	0.19861		

After 14 days of immersion

Long-term immersion accentuated brand differences (Table 4). Illusion continued to darken in coffee (mean ΔE=18) while stabilizing in cola and wine. Graphy showed its peak change in tea (mean ΔE=26) yet maintained low values (< 12) for the other beverages. Invisalign reached its maximum in wine (mean ΔE=12), whereas ClearCorrect again showed the greatest vulnerability to cola (mean ΔE=14). For every aligner, at least one beverage pushed ΔE well beyond the perceptibility threshold, confirming clinically relevant color degradation over two weeks.

**Table 4:** Comparison of discoloration of the aligners stained after 14 days of immersion

∆E After 14 Days of Immersion		N	Minimum	Maximum	Mean	Std. Deviation	F	p Value
Illusion	Tea	10	11.46	18.99	14.8000	2.56822	17.403	0.000
	Coffee	10	14.53	21.42	18.3530	2.95071		
	Cola	10	10.69	13.41	12.2310	0.92361		
	Wine	10	10.56	14.74	12.5480	1.46631		
Graphy	Tea	10	23.50	28.51	25.8700	1.76866	430.579	0.000
	Coffee	10	10.68	13.62	11.9270	1.21488		
	Cola	10	6.25	9.43	8.0060	0.98635		
	Wine	10	6.11	9.44	7.9380	1.06305		
Invisalign	Tea	10	6.02	9.30	7.8770	1.12128	44.722	0.000
	Coffee	10	8.27	11.30	10.3300	0.92597		
	Cola	10	6.21	8.93	7.4500	0.95303		
	Wine	10	11.00	13.15	11.6700	0.76868		
ClearCorrect	Tea	10	2.21	11.50	6.1130	2.94032	53.482	0.000
	Coffee	10	3.08	5.12	3.8840	0.57718		
	Cola	10	11.00	17.27	13.7460	2.09435		
	Wine	10	8.13	9.28	8.8310	0.35937		

Trajectory of color change

**Figure 3:**
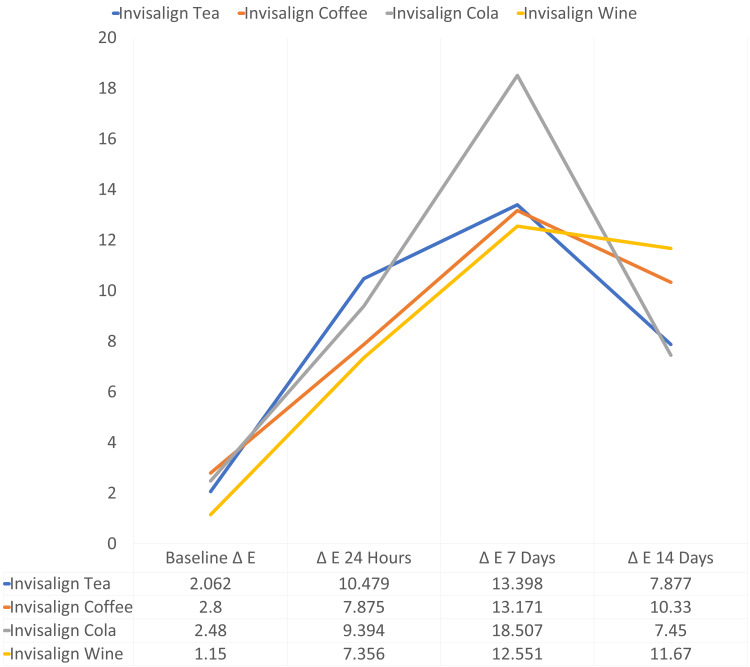
Comparison of the change in ΔE for Invisalign aligners immersed in different solutions

**Figure 4:**
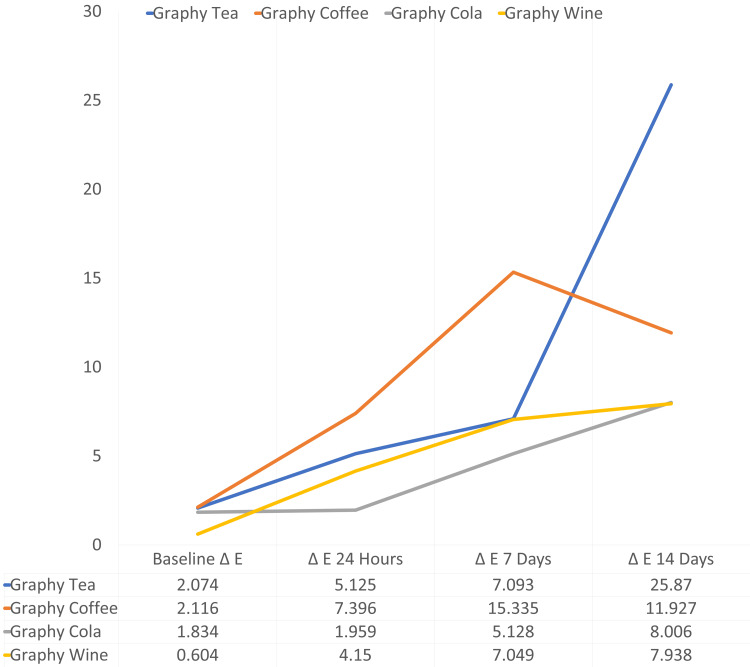
Comparison of the change in ΔE for Graphy aligners immersed in different solutions

**Figure 5:**
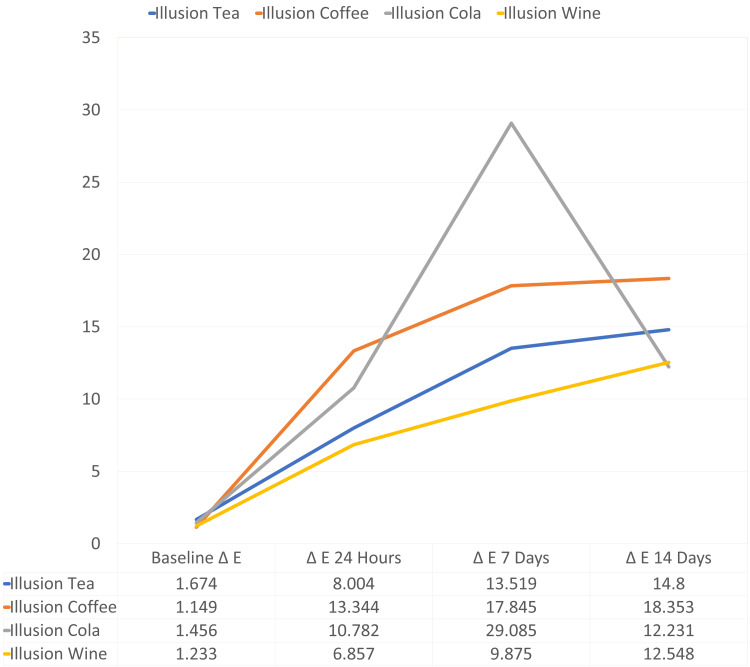
Comparison of the change in ΔE for Illusion immersed in different solutions

**Figure 6:**
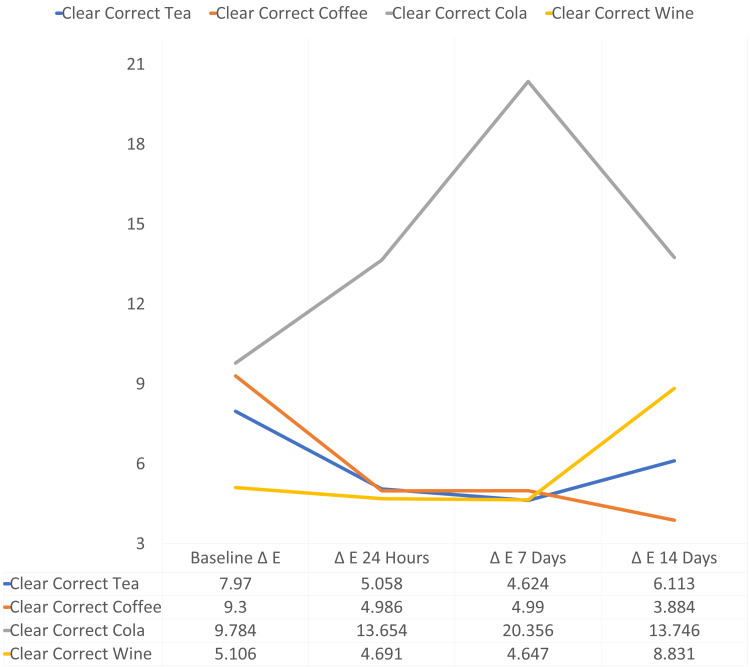
Comparison of the change in ΔE for ClearCorrect aligners immersed in different solutions

The mean ΔE trajectories from baseline to day 14 are presented as supplementary figures (Appendices Figures 3-6). Illusion and ClearCorrect displayed steep, beverage-dependent slopes, indicating rapid pigment uptake that plateaued or reversed after seven days. Graphy followed a shallow trajectory except in tea, where a late surge was evident. Invisalign exhibited the flattest profile, corroborating its moderate, broadly similar response to all drinks.

## Discussion

The present in vitro investigation evaluated and compared the color stability of four commercially available clear aligner materials when exposed to commonly consumed beverages, simulating the clinical scenario of continuous two-week wear. This design was chosen based on patient behavior studies, which reveal that nearly half of all aligner users fail to remove trays while drinking beverages other than water, thereby exposing the materials to repeated contact with staining agents [24]. The findings of the present study demonstrate that beverage composition, polymer chemistry, and immersion time are critical determinants of staining behavior, with complex time-dependent interactions influencing the extent and pattern of discoloration.

Coffee and cola emerged as the most potent chromogenic agents across all aligner brands, corroborating previous reports on their high staining potential in dental polymers. The chemical composition of these beverages explains their strong pigmenting ability. Coffee contains abundant polyphenolic compounds, including tannins and melanoidins, that can readily form hydrogen bonds with the polar functional groups of polymeric matrices [23,25]. Cola, by contrast, exerts a dual mechanism, wherein its acidic pH softens or partially hydrolyzes ester linkages in thermoplastic materials. At the same time, its caramel-based dyes diffuse into the polymer structure, leading to both extrinsic and intrinsic discoloration [26]. Red wine, though rich in anthocyanins and other chromophores, induced relatively milder staining. This could be attributed to the solubilizing effect of ethanol, which may interfere with pigment polymerization or reduce dye adherence to the polymer surface. Such beverage-dependent differences in staining behavior mirror findings from prior studies on orthodontic adhesives, ceramic brackets, and resin composites, underscoring that color instability is a universal concern across esthetic dental biomaterials [27].

Material composition and structure played an equally significant role in determining color change trajectories. Invisalign® (polyurethane-based) demonstrated moderate, beverage-independent ΔE shifts, consistent with the relatively balanced hydrophilic-hydrophobic nature of polyurethane chains. These polymers exhibit polar urethane groups that allow gradual sorption of chromogens, preventing abrupt pigment accumulation and producing a slow but steady color change [28-30]. In contrast, ClearCorrect™ (PETG) and Illusion® (multilayer polyester) showed rapid, pronounced discoloration, especially in cola and coffee, exceeding clinical perceptibility thresholds within 24 hours. The comparatively lower crystallinity of PETG, coupled with the presence of ester linkages susceptible to acid hydrolysis, facilitates faster ingress of pigments and acids into the polymer network [31]. The multilayer configuration of Illusion may further enhance pigment diffusion through interfacial layers, acting as microchannels for chromogen penetration.

Graphy TC-85®, the only directly printed aligner resin tested, exhibited a distinct pattern of behavior. While initially resistant to cola and coffee, it displayed a delayed but steep increase in ΔE when immersed in tea. This unique trajectory may be related to the hydrophilic polyphenols in tea, which gradually compromise the urethane-dimethacrylate cross-linked network of TC-85. Initially, the resin’s densely cross-linked structure resists water-based stains, but prolonged exposure to weak acids and tannins can lead to surface softening and breakdown of polymer chains, allowing chromogen entrapment [32]. Such findings indicate that while photocurable oligomers offer promising optical stability against acidic pigments, they may remain vulnerable to polyphenolic beverages that interact differently with the resin matrix.

Surface morphology also influenced the degree of staining. Thermoformed aligners, such as ClearCorrect™ and Illusion®, exhibit characteristic undulations, internal stresses, and microvoids that are created during heating and vacuum forming. These microstructural imperfections act as retention sites for pigments and facilitate light scattering, which amplifies perceived discoloration. Polyurethane aligners are prone to aging-related changes, such as microcrack formation and oxidation-induced yellowing, while PETG materials exhibit increased brittleness and surface roughness under acidic or thermal stress [33,34]. Such surface alterations further accelerate pigment adhesion and penetration, leading to greater variability in ΔE values across specimens.

The present results align closely with those of earlier investigations. Liu et al. reported that polyurethane aligners exhibited a significant color change in coffee compared to copolyester variants, whereas wine exerted minimal influence [34]. Similarly, Olteanu et al. demonstrated that a copolyester-elastomer hybrid material exhibited greater discoloration in cola and wine than PETG, emphasizing that polymer blends may not consistently outperform single-component materials [23]. Collectively, these studies highlight the absence of standardized formulations or manufacturing protocols for aligner polymers. Even subtle variations in monomer composition, thermal processing, or surface finishing can dramatically alter optical and sorptive properties, making inter-brand comparisons complex. The current findings thus contribute valuable data toward understanding how distinct polymer chemistries respond to real-world chromogenic challenges.

From a clinical standpoint, these findings underscore the need for a personalized approach to material selection and patient instruction. Although coffee is traditionally regarded as the leading cause of aligner discoloration, the results indicate that cola, a beverage widely consumed across all age groups, can be equally or even more deleterious, particularly for PETG-based systems. Illusion® and ClearCorrect™ surpassed the perceptibility threshold within the first day of exposure, suggesting that patients using these materials require stricter beverage restrictions or more frequent tray replacement. Conversely, Graphy TC-85® showed comparatively greater resistance to cola-induced discoloration under the present in vitro conditions. While such trends may inform hypotheses about material selection for patients with particular beverage habits, clinical preference should not be based solely on these laboratory data and requires confirmation in in vivo studies [35]. The polyurethane-based aligner, Invisalign®, demonstrated balanced performance across different beverages. Effective patient education on removing trays before drinking, regular cleaning with neutral solutions, and avoiding abrasive cleansers is imperative to maintain the esthetic appeal and functional longevity of aligners [24]. Nevertheless, such short-term laboratory findings cannot, on their own, justify selecting one aligner system over another for specific beverage habits and require confirmation in longitudinal clinical studies.

Despite its strengths, this study has several limitations inherent to itsin vitro design. The static immersion model does not fully replicate intraoral conditions where temperature fluctuations, salivary enzymes, mechanical abrasion, and biofilm deposition significantly influence staining dynamics. The absence of cyclic mechanical wear, brushing, and pH variations limits extrapolation to long-term clinical performance. Only four commercially available aligner systems were evaluated, which may limit generalizability to other brands and formulations. In addition, potential batch-to-batch or lot-to-lot variability in manufacturing could affect polymer properties and staining behavior, and this was not independently assessed. Surface topography and roughness (e.g., via scanning electron microscopy (SEM) or profilometry) were not measured, so the contribution of microscopic surface features to pigment retention could not be directly quantified. Manufacturing and model-printing processes have been shown to influence dimensional accuracy and material behavior in clear aligner workflows [15], and our findings should be interpreted in this context. Future research should incorporate dynamic immersion models, simulated mastication, and exposure to microbial biofilms to better mimic the oral environment. Studies exploring mechanical degradation, tensile strength, and post-staining surface morphology using advanced imaging and spectroscopy techniques could also yield more profound insights into the interplay between chemical and physical deterioration.

Overall, beverage-induced staining of clear aligners is a multifactorial phenomenon governed by the complex interplay of polymer chemistry, beverage composition, and exposure duration. The present study reinforces that color stability varies significantly among aligner systems. PETG and multilayer polyester blends show rapid discoloration, polyurethane displays moderate, uniform changes, and photocurable resins exhibit beverage-specific delayed effects. Understanding these material-beverage interactions is crucial for clinicians to make evidence-based decisions, guide patient behavior, and encourage the development of next-generation stain-resistant aligner materials. Such knowledge ultimately translates into enhanced patient satisfaction, sustained esthetic outcomes, and improved clinical success in clear aligner therapy.

## Conclusions

The present study demonstrated that the color stability of clear aligner materials is highly dependent on both the polymer composition and the staining beverage. PETG and multilayer polyester blends showed rapid and pronounced discoloration in cola and coffee, while polyurethane exhibited moderate changes, and the TC-85 resin displayed delayed staining in tea. These findings underscore the importance of aligning material selection with patient beverage habits and reinforcing hygiene protocols to preserve the aesthetic integrity of aligner therapy. However, the findings should be interpreted as short-term in vitro evidence and integrated cautiously with clinical data on clear aligner effectiveness and patient outcomes.
